# Airway management using laryngeal mask airway (LMA) in a patient in a lateral decubitus position: A case report

**DOI:** 10.1097/MD.0000000000018287

**Published:** 2019-12-20

**Authors:** Jung A Lim, Min Yeong Jeong, Jong Hae Kim

**Affiliations:** Department of Anesthesiology and Pain Medicine, School of Medicine, Daegu Catholic University, Daegu, Republic of Korea.

**Keywords:** airway management, hematoma, intubation, laryngeal masks, neurofibromatosis 1

## Abstract

**Rationale:**

Airway management of patients in a lateral decubitus position (LDP), who cannot lie supine is challenging for anesthesiologists. In a previous study, laryngeal mask airway (LMA) was found to be superior to conventional endotracheal intubation in LDP.

**Patient Concerns:**

A 38-year-old man diagnosed with type I neurofibromatosis presented with pain caused by a large hematoma (28 × 8 cm) located in the left upper back. On arrival at the operating theater, he was in a right LDP because of the aggravation of pain in the supine position.

**Diagnoses:**

Laryngoscopy-guided endotracheal intubation was expected to be difficult in LDP.

**Interventions:**

After the induction of anesthesia, a non-inflatable LMA was introduced into the laryngopharynx with the patient in LDP. He was then maneuvered into a supine position and removal of the LMA was followed by endotracheal intubation.

**Outcomes:**

The surgery for the removal of the hematoma was performed in a prone position. The airway intubated with an endotracheal tube was well maintained during the entire surgery.

**Lessons:**

LMA is a useful device for airway management in patients in LDP who cannot lie supine.

## 1 Introduction

Airway management of patients in a lateral decubitus position (LDP) is required in uncommon clinical situations such as severe posterior neck and back mass precluding a supine position, accidental extubation,^[1]^ or when regional anesthesia is determined to be inadequate during surgery in LDP. Via a simple change in position (supine to lateral), a functioning airway can become compromised.^[2]^ Similarly, the management of a difficult airway can become more challenging.

A difficult airway can be managed via an intubating laryngeal mask airway (LMA),^[3,4]^ videolaryngoscopy,^[2]^ fiberoptic intubation,^[5,6]^ light-wand-assisted intubation,^[7]^ and an LMA without intubation.^[8]^ The use of an LMA is the most simple and causes the least discomfort because the only necessary process is insertion into the laryngopharynx. In addition, as a supraglottic airway device LMA is included in the Difficult Airway Society guidelines.^[9]^ In the present case, we inserted an LMA into the laryngopharynx after induction of anesthesia to secure the airway of a patient who was in LDP due to pain caused by hematoma developing from an upper back mass. The patient was then placed in a supine position and the LMA was replaced with an endotracheal tube.

## 2 Case

**Figure 1:**
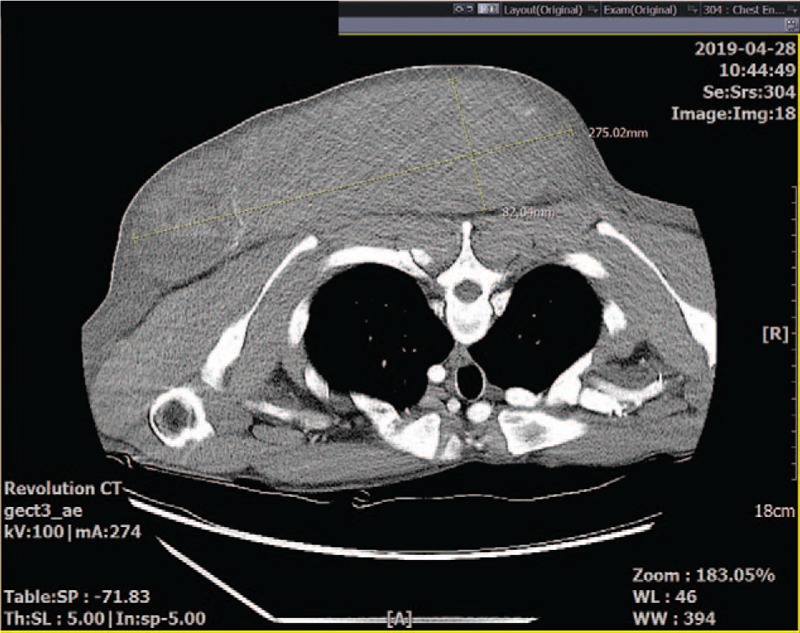
Preoperative chest computed tomography depicting a huge mass of 28 cm in width and 8 cm in height in the left upper back.

A 38-year-old man, 166 cm in height and 62 kg in weight, with type I neurofibromatosis presented with pain associated with a large mass in the upper back region. He reported that the mass had been growing gradually since being excised 8 years prior. One day before the current admission, pain had begun to emanate from the mass, which had gradually increased to 7 cm in diameter. Its visible diameter on the surface reportedly kept increasing, and it was 15 cm at the time of the current admission. Preoperative enhanced chest computed tomography revealed a huge mass of 28 cm in width and 8 cm in height (Fig. 1).

On physical examination, the mass was hard and exhibited heat and tenderness on palpation. Pain from the mass prevented the patient from lying supine, but neck motion was not restricted. Complete blood cell count on admission revealed hemoglobin 13.0 g/dL, platelet count 255,000/μL, and leukocyte count 11,500/μL. These parameters had abruptly changed to hemoglobin 6.6 g/dL, platelet count 135,000/μL, and leukocyte count 16,700/μL by 21 hours after admission, and his respective systole, diastole, and heart rate had changed from 120/70 mmHg and 78 beats/min to 130/80 mmHg and 160 beats/min. Due to suspected active bleeding from the mass, emergent evacuation of the hematoma was planned.

The patient was admitted to the operating theater in right LDP. A pad was placed beside the right side of his head to maintain it in a neutral position. Electrocardiography and pulse oximetry were continuously monitored. The right radial artery was catheterized under ultrasound guidance for continuous monitoring of arterial blood pressure and frequent arterial blood sampling. During catheterization, the patient was preoxygenated with 100% oxygen, and a non-inflatable LMA (size 4 i-gel^TM^, Intersurgical Ltd., Wokingham, Berkshire, UK) was prepared by lubricating the back, side, and front of the cuff with its orifice spared.

**Figure 2:**
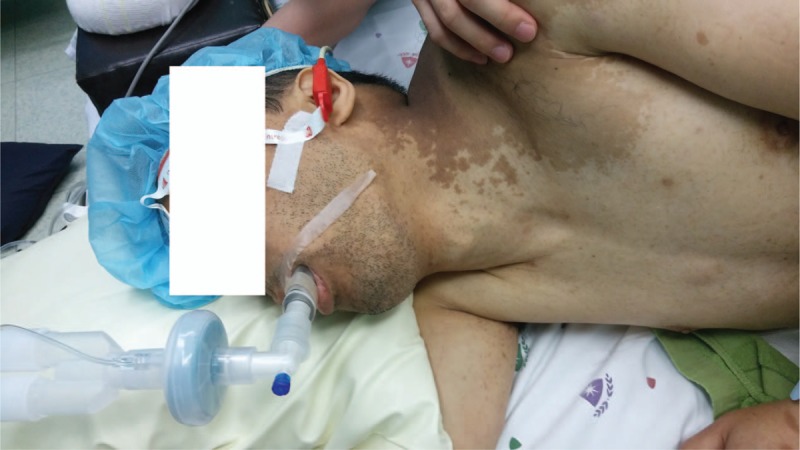
The non-inflatable laryngeal mask airway (size 4 i-gel^TM^, Intersurgical Ltd., Wokingham, Berkshire, UK) inserted into the patient placed in a right lateral decubitus position.

Anesthesia was then induced via 70 mg ketamine and 3 mg midazolam. In conjunction with the loss of consciousness 70 mg rocuronium was administered to facilitate the insertion of the prepared LMA, and the patient was manually ventilated via a mask. After the train of four count became 0, the LMA was inserted into the laryngopharynx. During its insertion an assistant placed the patient's head in the sniffing position with the head extended and the neck flexed. The anesthesiologist gently introduced the LMA downwards and backwards along the hard palate until resistance against insertion was encountered (Fig. 2). After the LMA was inserted the patient was manually ventilated to confirm appropriate placement of the LMA based on detection of end-tidal carbon dioxide from the exhaled gas via capnography. The position of the LMA was secured by taping its integral bite block to the facial skin. The lungs were mechanically ventilated with 50% oxygen. The tidal volume and respiratory rate were adjusted to maintain an end-tidal carbon dioxide concentration between 35 and 40 mmHg and a peak airway pressure below 20 cm H_2_O. Anesthesia was maintained with sevoflurane to maintain state entropy values between 40 and 60.

**Figure 3:**
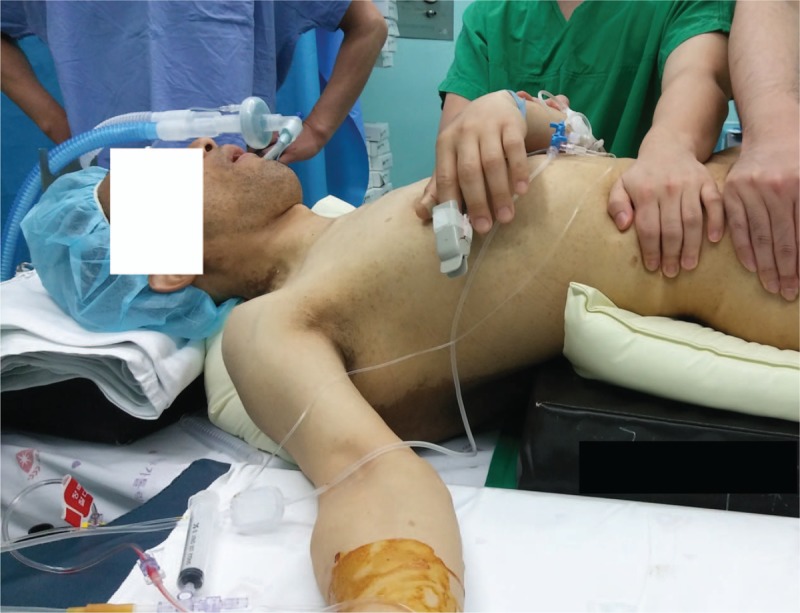
The patient placed in a supine position with pillows and pads applied to protect the mass from compression between the patient's body and the operating table. The patient was intubated after removal of the laryngeal mask airway.

**Figure 4:**
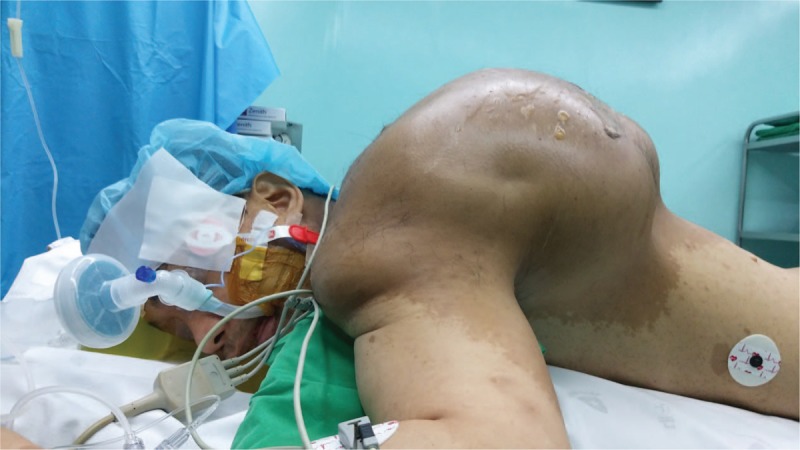
The patient placed in a prone position for hematoma removal.

At the conclusion of the LMA insertion the patient was placed in a supine position. Pads and pillows were placed behind the head and the body trunk below the mass in order that the mass not be compressed while he was in the supine position (Fig. 3). After the LMA was removed from the mouth, the trachea was intubated with a 7.5-mm internal diameter cuffed reinforced endotracheal tube using a portable videolaryngoscope (UEscope, UE Medical Devices Inc., Newton, MA). The capnographic waveform confirmed the placement of the tube into the airway. Auscultation of breath sounds in both lungs ruled out endobronchial placement of the tube. Mechanical ventilation was maintained as described above. Under ultrasound guidance the right internal jugular vein was catheterized to administer blood products and fluids and to monitor central venous pressure continuously. The patient was then placed in a prone position with the head turned to the left (Fig. 4).

The dependent eye was protected from external compression. Arms were not abducted greater than 90°. Every pressure point was padded. Endotracheal tube positioning, adequate ventilation, and patency of the arterial and venous lines were reassessed after instigation of the prone position. The surgery took 100 minutes. During the surgery the patient received 900 mL of packed red blood cells, 640 mL of fresh frozen plasma, 2890 mL of plasmalyte, and 50 mL of 6% hydroxyethyl starch 130/0.4 to compensate for blood loss. He was also administered 10 mg ephedrine, 20 μg norepinephrine, and 600 mg CaCl_2_. At the end of the surgery the patient could be placed in a supine position and the neuromuscular blockade maintained by a total of 100 mg rocuronium was reversed via 200 mg sugammadex.

**Figure 5:**
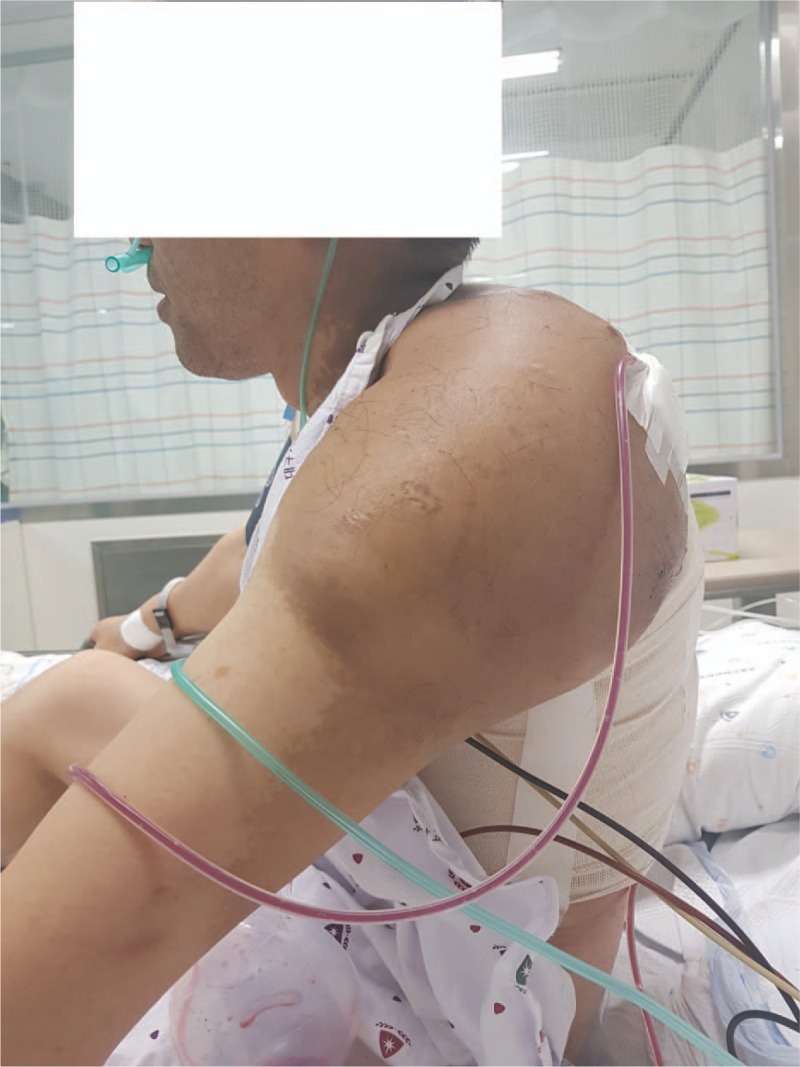
The conscious patient in a sitting position after removal of the hematoma.

After recovery of spontaneous breathing and consciousness, the endotracheal tube was withdrawn uneventfully. The patient was transferred to the surgical intensive care unit. On the first postoperative day, he was discharged to the general ward and was satisfied with the results of the surgery (Fig. 5). The remnant mass was excised 1 month after the surgery, and at that time-point, the patient had experienced no complications related to the preceding above-described surgery. The patient has provided informed consent for the publication of this case report.

## 3 Discussion

In the present case, the use of a non-inflatable LMA facilitated easy and simple airway management of the patient in LDP who found it too painful to adopt a supine position. To anesthetize the patient, who was hypovolemic and needed to undergo surgery in a prone position, general anesthesia with mechanical ventilation via an endotracheal tube under neuromuscular blockade was indicated. However, direct laryngoscopy-guided endotracheal intubation of a patient in the prone position is nearly impossible. Although endotracheal intubation of a patient in LDP is theoretically easier than that of a patient in a prone position, most anesthesiologists are not familiar with the procedure due to a lack of experience arising from the very low number of potentially applicable cases.

As does the supine position, the LDP enables access to the airway. Notably, however, the use of techniques to facilitate direct laryngoscopy-guided endotracheal intubation is limited. A sniffing position achieved via head extension at the atlanto-occipital joint and flexion at the lower cervical joint^[10]^ aligns the oral, pharyngeal, and laryngeal axes, facilitating successful endotracheal intubation. Before instigating the position, the head should be oriented neutrally (no lateral bending or rotation of the head). A supine position permits both a neutral head orientation and a sniffing head position. Regrettably, the present patient was in LDP because he could not be placed in a supine position due to pain derived from an upper back mass. Thus, a pad was placed below the dependent side of the head to orient the head in a neutral position and then achieve a sniffing position. However, an LMA was inserted instead of an endotracheal tube because only a sniffing position does not guarantee successful endotracheal intubation.

Another technique to facilitate direct laryngoscopy-guided endotracheal intubation is the application of backward, upward, and right-sided pressure on the thyroid and cricoid cartilages (the “BURP” maneuver), which provides better glottic exposure. Because an operating table exerts pressure against BURP, performing BURP is feasible in a supine position. Pressure against BURP is absent in LDP however, thereby preventing the performance of optimal BURP. Therefore, the use of an LMA is justified in this case because BURP maneuver is not necessary to use an LMA.^[11]^

The side of LDP has substantial effects on the success rate of laryngoscopy-guided endotracheal intubation. When a patient is placed in a right LDP, the anesthesiologist should insert their right hand holding an endotracheal tube into the space between the right side of the face and the operating table, which is limited due to the right LDP. The right placement of the tongue resulting from a right LDP can also impede the introduction of an endotracheal tube.^[2]^ It has been reported that even left LDP can compromise the laryngoscopic view, leading to longer performance time and higher endotracheal intubation failure rates compared to LMA insertion.^[8]^ In addition, endotracheal intubation through an intubating LMA had a success rate close to 100% regardless of the side of LDP.^[4]^ For this reason, the use of an LMA was indicated in the present patient in a right LDP.

Among various types of LMAs, we elected to use a non-inflatable LMA (i-gel^TM^) because its soft non-inflatable cuff makes its use easy and reliable and does not affect the airway patency which can be compromised by the tongue displaced by inappropriate inflation of an inflatable cuff. In addition, changing from supine to LDP does not affect the oropharyngeal leak pressure and position in the laryngopharynx.^[12]^ However, the use of LMAs should be discouraged unless oral, pharyngeal, and laryngeal axes can be aligned.^[13]^

When a videolaryngoscope [the Airway Scope (Pentax, Tokyo, Japan)] was used to intubate patients, no differences in success rate of endotracheal intubation were found between right LDP, left LDP, and supine position despite the direct laryngoscopic view worsened by LDP.^[2]^ Because the insertion of the blade (Intlock) of the Airway Scope delivers an endotracheal tube to the glottic opening, the limited space between the right side of the face and an operating table minimally affected the operators’ performance thereby producing the comparable success rate between two LDPs. However, in this case, we intubated the patient using UEscope in the supine position, because the absence of Intlock in the UEscope makes an operator insert the right hand holding an endotracheal tube into the space limited by right LDP between the right side of the face and an operating table.

Fiberoptic bronchoscope-guided endotracheal intubation seems to be the best way to manage the airway for patients in LDP due to shorter time to endotracheal intubation (33 seconds in median) and higher first-attempt intubation success rate (97%) compared to the supine position.^[6]^ However, successful endotracheal intubation within one minute under fiberoptic bronchoscope guidance requires considerable expertise^[14]^ compared to videolaryngoscope^[15]^ and LMA^[16]^. Particularly, the incompetence in manipulating the bronchoscope might delay endotracheal intubation causing hypoxia in patients with neuromuscular blockade under general anesthesia. Therefore, the general use of fiberoptic bronchoscope should be limited in LDP. In addition, light-wand and intubating LMA also can be used for endotracheal intubation. However, esophageal intubation cannot be completely avoided.^[3,7]^

In conclusion, considering the limitations imposed by the LDP and other airway management techniques, the use of an LMA in LDP followed by its removal and subsequent endotracheal intubation in the supine position is highly recommended for airway management in patients in LDP who cannot lie supine.

## Author contributions

**Data curation:** Jung A Lim, Min Yeong Jeong, Jong Hae Kim.

**Supervision:** Jong Hae Kim.

**Writing – original draft:** Jung A Lim, Min Yeong Jeong, Jong Hae Kim.

**Writing – review & editing:** Jung A Lim, Jong Hae Kim.

Jung A Lim orcid: 0000-0002-7427-5483.

Jong Hae Kim's orcid: 0000-0003-1222-0054.
